# Arrhythmogenic Implications of Inflammatory Bowel Disease: Its Mechanisms and Treatment Effects

**DOI:** 10.7759/cureus.99503

**Published:** 2025-12-17

**Authors:** Enayat S Almasri, Tabish W Siddiqui, Rama M Almasri, Gizalla Abdulla, Angela A Benoj, Gayathri Pradeep, Mais O Abu-Sa'da, Ahmad Alaboud, Rand N Fatayerji, Raqshan W Siddiqui

**Affiliations:** 1 Department of Medicine, Emirates Health Services, Ras Al Khaimah, ARE; 2 Department of Medicine, Emirates Health Services, Fujairah, ARE

**Keywords:** atrial fibrillation, cardiovascular diseases, crohn disease, inflammation, inflammatory bowel disease, ulcerative colitis

## Abstract

Inflammatory bowel disease, comprising Crohn’s disease and ulcerative colitis, is increasingly recognized as a systemic condition that extends beyond the gastrointestinal tract. Growing evidence indicates a significant association between inflammatory bowel disease and cardiovascular complications, particularly cardiac arrhythmias, driven by persistent inflammation and, in some cases, medication effects. This review integrates current clinical and mechanistic insights into the link between inflammatory bowel disease and arrhythmia risk. Recent epidemiological and genetic studies consistently show an elevated incidence of atrial fibrillation, atrioventricular block, and other conduction abnormalities in affected patients, independent of traditional cardiovascular risk factors. Systemic cytokine activation, autonomic dysfunction, and structural cardiac remodeling appear to be central mediators. Drug effects vary, with aminosalicylates occasionally causing reversible myocarditis, corticosteroids producing dose and duration-related cardiovascular effects, and biologic agents generally demonstrating cardiac safety. Recognizing these associations highlights the importance of comprehensive cardiovascular assessment and inflammation control in patient care. Coordinated multidisciplinary management and long-term research are essential to refine risk prediction and improve cardiac outcomes in this population.

## Introduction and background

Inflammatory bowel disease (IBD) is a chronic immune-related illness, defined as a relapsing and remitting inflammatory disorder of the gastrointestinal tract, that constitutes a major and growing global public health concern [1]. Crohn’s disease (CD) and ulcerative colitis (UC) are the two main types of IBD. Once thought to be mainly a disease of Western countries, IBD is now increasingly recognized in Asia, the Middle East, and other newly industrialized areas [2]. This change shows a global shift in disease patterns. Rapid urbanization, environmental changes, and lifestyle changes, especially in developing regions, are driving this trend [2]. Globally, the number of people with IBD has steadily increased [3]. Estimates suggest that nearly seven million individuals are now living with the disease [3]. Predictive models show that the number of cases will increase over the next decade [2,3]. East Asia and other developing regions where rapid health changes are occurring will be the primary locations for this increase [2]. In contrast, Western countries have mostly entered a phase of increasing prevalence [2]. This phase features stable incidence rates, but the overall disease burden is rising due to patients living longer [2]. IBD also creates a significant and growing economic burden [4]. In high-income countries, annual out-of-pocket costs for medical care, including hospital stays, outpatient visits, and prescription drugs, can range from $9,000 to $12,000 per patient [4]. This substantial financial impact emphasizes how crucial it is to address the issue of IBD by taking into account both the growing worldwide burden and related healthcare expenses.

IBD arises from a complex interaction of genetic susceptibility, immune system dysregulation, and environmental influences, causing persistent, recurring intestinal inflammation [1,5]. At the cellular level, this inflammation undermines the integrity of the intestinal epithelial barrier and encourages prolonged immune activation, frequently indicated by the presence of proinflammatory epithelial metaplastic cells in the gut mucosa [5]. Although the main site of IBD symptoms is the intestines, the immunological activation that occurs may affect other parts of the body, involving several organ systems [6]. Systemic inflammation, referring to the persistent elevation of circulating cytokines and immune mediators, in IBD is gaining attention for its deleterious effects on heart function and the development of abnormal heart rhythms [6]. This occurs through cytokine-mediated disruptions in myocardial tissue and the cardiac conduction system, autonomic dysfunction, and structural remodeling of the myocardium [6]. These extraintestinal effects highlight the broader systemic burden of IBD and the importance of recognizing its potential multi-organ involvement [6]. Evidence indicates that IBD may be associated with systemic consequences, including cardiovascular involvement, underscoring the need to view IBD as a multisystem disease [6].

Standard therapeutic strategies for IBD typically begin with corticosteroids such as budesonide or prednisone for managing moderate to severe flares [7]; 5-aminosalicylates (5-ASA), such as mesalazine or sulfasalazine, are primarily used in UC. For patients with refractory or more severe disease, advanced treatments include biologic agents such as tumor necrosis factor (TNF) inhibitors and interleukin (IL) antagonists, as well as newer small molecule therapies [7]. These advanced options aim to enhance drug delivery, reduce adverse effects, and improve long-term disease control and patient outcomes. Importantly, established treatments have also been associated with a modest but recognized risk of arrhythmias, particularly atrial fibrillation (AF) [8]. While the systemic inflammation characteristic of active IBD remains a major contributor to arrhythmogenic risk, the potential cardiac effects of these therapies themselves should not be overlooked. Careful cardiovascular monitoring may be warranted, especially in patients with preexisting risk factors [8].

Despite evidence indicating an increased risk of arrhythmias in patients with IBD, the extent to which this risk is driven by the underlying disease versus its treatments remains unclear. Systemic inflammation associated with active IBD is known to contribute to arrhythmogenesis, while emerging data suggest that therapies such as biologics, immunomodulators, and corticosteroids may also carry an independent, though modest, arrhythmogenic potential [6,8]. However, current research is limited by inconsistent findings, a lack of treatment-stratified analyses, and inadequate control for disease activity, making it difficult to disentangle the relative contributions of disease-induced and treatment-induced risk. Given the clinical importance of both cardiovascular complications and effective IBD management, further investigation is urgently needed to clarify these relationships [3]. A deeper understanding will enable clinicians to better identify patients at highest risk, optimize treatment regimens that balance inflammation control with cardiovascular safety, and develop targeted monitoring protocols. This review aims to elucidate the interplay between IBD-related systemic inflammation, therapeutic exposures, and the development of cardiac arrhythmias, providing a comprehensive synthesis of the multifactorial mechanisms that contribute to arrhythmia risk in this population. By integrating evidence on disease-driven and treatment-related pathways, this review seeks to refine current understanding and support the development of more effective prevention, surveillance, and management strategies for cardiovascular complications in patients with IBD.

## Review

Materials and methods

Search Strategy and Study Selection

This review adhered to the Preferred Reporting Items for Systematic Reviews and Meta-Analyses (PRISMA) guidelines. A comprehensive search was performed across major electronic databases, including PubMed/MEDLINE, SCOPUS, Embase, and Google Scholar. The search utilized a combination of Medical Subject Headings (MeSH) and free-text terms combined with Boolean operators (AND, OR) to maximize sensitivity and specificity.

Key search terms included: "Inflammatory Bowel Disease" OR "Crohn's Disease" OR "Ulcerative Colitis" AND "Arrhythmia" OR "Atrial Fibrillation" OR "Ventricular Tachycardia" AND "IBD treatment" OR "biologics" OR "corticosteroids" OR "JAK inhibitors."

The full search strings for each database are provided in Appendix 1, ensuring that the search strategy is fully reproducible. The search was limited to English-language studies published between 2000 and 2025, with the final search conducted on September 25, 2025.

Initial database queries returned 380 articles. After removing 57 duplicates, 323 records remained. Following title and abstract screening, 198 articles were excluded due to irrelevance or failure to meet the study type criteria, leaving 125 full-text articles for detailed assessment.

Inclusion and Exclusion Criteria

The inclusion criteria targeted original, peer-reviewed research that provided quantitative risk estimates (hazard ratio (HR) or odds ratio (OR)) for the association between IBD or its treatments and specific cardiac arrhythmias. Studies had to clearly assess the risk in relation to either the underlying disease (systemic inflammation) or specific IBD pharmacological treatments. Non-English studies and those lacking statistical analysis were excluded, as were non-peer-reviewed studies, such as conference abstracts or editorials. During the full-text review, 114 articles were excluded for lack of robust statistical analysis (n = 41), insufficient sample size/non-representative data (n = 36), or focusing on nonspecific cardiovascular outcomes (n = 37). A final selection of 11 studies met all criteria.

Quality and Risk of Bias Assessment

The methodological quality and risk of bias of observational studies were systematically assessed using the Newcastle-Ottawa Scale (NOS). Each study was evaluated across three domains:

1. Selection of study groups: representativeness of the exposed cohort, selection of the non-exposed cohort, ascertainment of exposure, and demonstration that the outcome of interest was not present at baseline.

2. Comparability of cohorts: assessment of whether the study controlled for key confounders such as age, sex, disease activity, or comorbidities.

3. Outcome assessment: how outcomes were measured (e.g., validated diagnostic criteria and clinical records), duration and adequacy of follow-up, and adequacy of outcome ascertainment.

Scoring: Studies could achieve a maximum of nine points. Studies with NOS ≥ 6 were considered moderate to high quality and prioritized in the synthesis.

**Figure 1:**
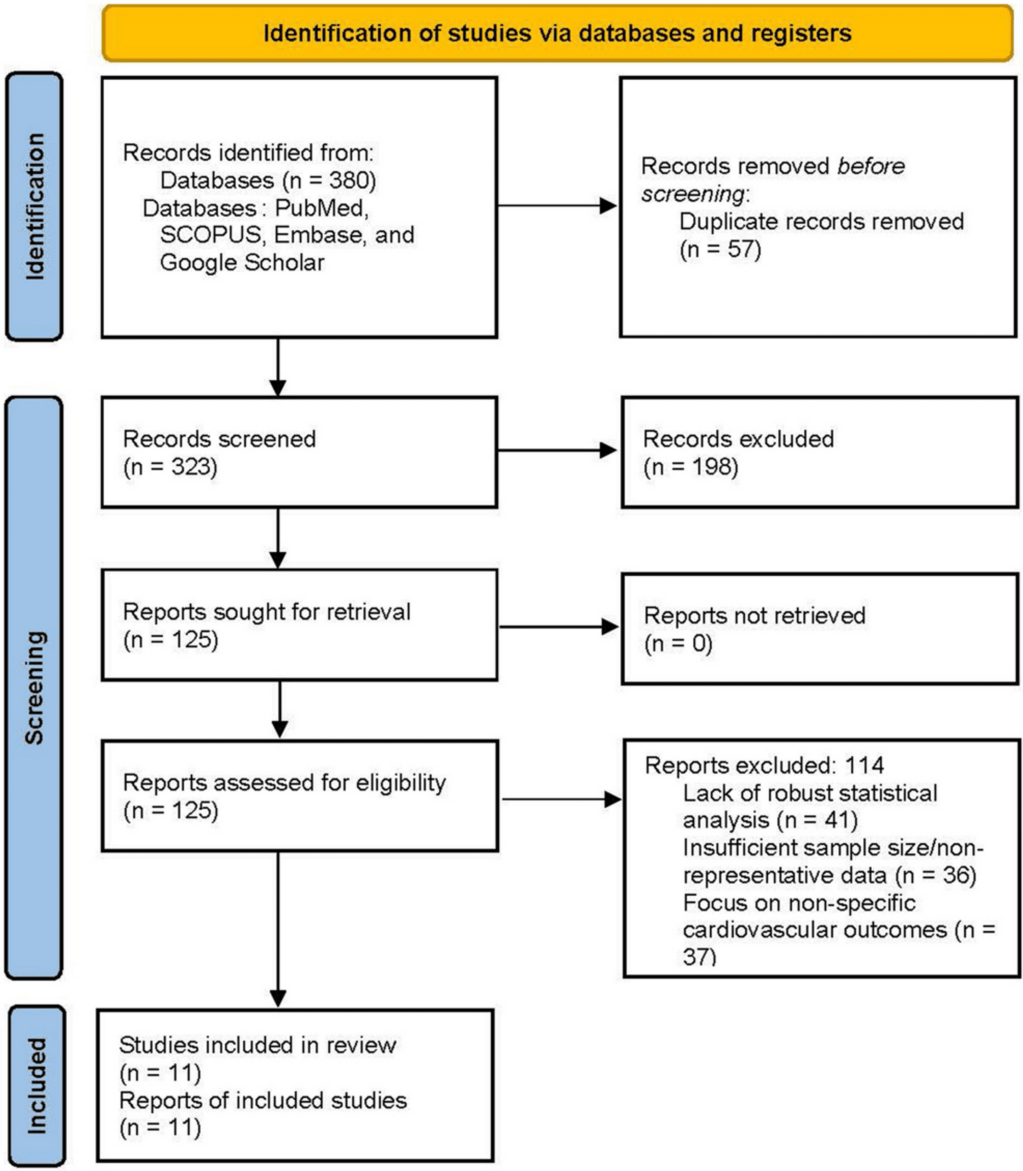
PRISMA flowchart of selected studies PRISMA: Preferred Reporting Items for Systematic Reviews and Meta-Analyses.

The entire selection process is illustrated in the PRISMA flowchart (Figure 1).

Data Extraction and Synthesis

Data extraction was conducted using a standardized form, capturing the study design, population size, IBD subtype, specific exposure, arrhythmia outcome, and key quantitative findings (HRs/ORs with 95% confidence interval (CI)).

Extracted information included study characteristics (study design, country/region, setting, sample size, follow-up duration, and IBD subtype), exposures and comparators (disease-related exposures such as systemic inflammation, active disease, and genetic predisposition; treatment-related exposures such as corticosteroids, biologics, immunomodulators, and JAK inhibitors), outcome data (type of arrhythmia and method of outcome ascertainment), quantitative results (HR, OR, relative risks (RR) with 95% CI, p-values, and adjustment for confounders), and study quality/risk of bias (NOS score and key methodological considerations).

Given the significant heterogeneity among included studies, formal quantitative meta-analysis was not feasible. This heterogeneity arose from multiple sources, including differences in study design (cohort, case-control, and registry-based studies), population characteristics (age distribution, disease duration, geographic location, and comorbidities), outcome definitions (clinical diagnosis, electrocardiography (ECG), or administrative codes, with varying follow-up durations), exposure assessment (diverse IBD subtypes, disease activity measures, and treatment regimens), and reported effect measures (HR, OR, RR, with differing levels of confounder adjustment). Pooling these data quantitatively could therefore produce misleading or biased results.

Instead, a structured narrative synthesis was conducted using the following stepwise approach:

1. Thematic grouping: Studies were categorized based on the IBD disease effect (systemic inflammation, active disease, and genetic predisposition) and the IBD therapy effect (corticosteroids, biologics, immunomodulators, and JAK inhibitors).

2. Effect size reporting: HR, OR, and RR with corresponding 95% CI were extracted and reported; p-values were included when available.

3. Cross-study comparison: Findings were compared across studies to identify consistencies, discrepancies, and potential patterns in arrhythmia risk.

4. Qualitative appraisal: Study quality, methodological rigor, and potential confounders were incorporated into the synthesis to contextualize quantitative findings.

Key findings, effect sizes, and risk profiles were summarized in tables. The final list of included studies, along with their NOS score, is presented in Table 1.

**Table 1:** Final list of articles included in the review IBD: Inflammatory bowel disease; RCT: Randomized controlled trial; NOS: Newcastle-Ottawa Scale.

Study	Design	Thematic Group	Population / Sample	NOS Score
Shu and Huang, 2025 [9]	Genetic phenotype analysis	IBD disease effect	34,652 IBD; >300k controls	7
Narous et al., 2025 [10]	Retrospective cohort study	IBD disease and therapy effect	10,992 IBD vs 102,875 controls	8
Sun et al., 2023 [11]	Sibling-controlled cohort study	IBD disease effect	83,877 IBD + matched siblings	9
Aniwan et al., 2018 [12]	Population-based cohort	IBD disease effect	736 IBD vs 1,472 controls	8
Zhou et al., 2023 [13]	Meta-analysis of RCT	IBD therapy effect	14,442 surgical patients	6
Larsen et al., 2023 [14]	Nationwide population-based cohort study	IBD therapy effect	44,053 IBD	9
Nguyen et al., 2022 [15]	Worldwide observational cross-sectional study	IBD therapy effect	>21 million reports	6
Chen et al., 2022 [16]	Systematic review	IBD therapy effect	52 IBD cases	6
Liu et al., 2021 [17]	Meta-analysis of RCTs	IBD therapy effect	13,803 surgical patients	6
Pujades-Rodriguez et al., 2020 [18]	Population-based cohort study	IBD therapy effect	87,794 autoimmune patients (27,739 IBD)	8
Halonen et al., 2007 [19]	Double-blind randomized multicenter trial	IBD therapy effect	241 surgical patients	7

Diagnosing IBD

Accurate diagnosis of IBD mandates a rigorous stepwise protocol combining clinical, endoscopic, histopathological, laboratory, and imaging parameters to distinguish CD and UC from each other and mimicking conditions [1]. A detailed history and thorough physical examination remain the cornerstone of initial assessment, providing essential clues such as the pattern and duration of symptoms, extraintestinal manifestations, and risk factors [1]. In UC, patients often present with persistent or recurrent mucous or bloody stools, increased stool frequency, and abdominal pain, whereas CD frequently manifests with chronic abdominal pain, diarrhea (sometimes bloody), weight loss, fever, and perianal lesions [1].

Laboratory evaluation plays a supportive but critical role. Routine testing includes complete blood counts, stool cultures, infection screening, fecal calprotectin, erythrocyte sedimentation rate (ESR), and C-reactive protein (CRP), which help exclude infectious etiologies and quantify systemic inflammation [1]. Serologic markers, including anti-*Saccharomyces cerevisiae* antibodies (ASCA) and perinuclear antineutrophil cytoplasmic antibodies (pANCA), can aid in distinguishing CD and UC, though no single laboratory test is diagnostic [1].

Endoscopy with biopsy remains indispensable for direct visualization and histopathological confirmation [20]. UC typically presents with continuous mucosal inflammation beginning in the rectum, granularity, superficial ulceration, and pseudopolyps, whereas CD demonstrates discontinuous, segmental, transmural lesions with deep longitudinal ulcers, cobblestoning, strictures, and fistulas [20]. Histopathology in UC shows diffuse mucosal inflammatory infiltrates, crypt abscesses, goblet cell depletion, and chronic changes such as gland distortion and atrophy, while CD often exhibits transmural inflammation with noncaseating granulomas, fissures, and fistulas [20]. Biopsy interpretation, together with clinical and endoscopic findings, is crucial for accurate phenotyping and management [20].

Standardized scoring systems are central to the assessment and management of IBD. The Mayo Clinic Score and the Simple Clinical Colitis Activity Index (SCCAI) are two of the most commonly utilized measures in UC [21]. These indices take into account clinical characteristics such as the frequency of bowel movements, rectal bleeding, endoscopic results, and the physician’s global assessment (PGA) [22]. Using a scale from normal to severe, the PGA is a clinician's overall assessment of disease activity that combines objective subscores, physical examination results, symptom diaries, and occasionally laboratory or imaging results [22]. The SCCAI, in particular, is well validated and has shown strong correlation with both endoscopic outcomes and noninvasive biomarkers [21]. The Crohn's Disease Activity Index (CDAI) and the Harvey Bradshaw Index (HBI), which emphasize abdominal pain, overall health, the existence of complications, and pertinent laboratory markers, are the most widely used tools for measuring disease activity in CD [23]. These established indices, particularly SCCAI, CDAI, and HBI, have been shown in multiple studies to have a strong correlation with fecal calprotectin and CRP, two reliable noninvasive markers of intestinal inflammation [23]. The Inflammatory Bowel Disease Index (IBDEX), a more contemporary grading system, was created to provide a single severity metric for both CD and UC [22]. Although preliminary findings indicate that it correlates with current indices and biomarkers, there is currently little data supporting its widespread validation and clinical adoption, and major guidelines do not support it [22,23]. Overall, the Mayo Score, SCCAI, CDAI, and HBI continue to serve as the most established and reliable instruments for stratifying disease activity, guiding therapeutic decisions, and supporting longitudinal patient monitoring in both clinical practice and research.

Advancing technology has significantly expanded the diagnostic spectrum for IBD. In UC, imaging plays a limited diagnostic role. However, a barium enema may demonstrate the classic lead-pipe colon appearance in chronic disease [1,24]. In CD, magnetic resonance enterography (MRE) and computed tomography enterography (CTE) are validated tools with reported sensitivities of around 90% or higher for detecting active small bowel inflammation [24]. These imaging modalities complement conventional endoscopy, which has limited reach into the small bowel, and they provide additional value by identifying complications such as strictures, fistulas, and penetrating disease that may not be visible endoscopically [1,24]. Recent developments in magnetic resonance imaging (MRI), such as techniques for differentiating between inflammation and fibrosis, noncontrast diffusion-weighted imaging, and quantitative MRI parameters for evaluating treatment response, improve prognostication and diagnostic accuracy [24].

Current consensus emphasizes a multidisciplinary, integrated approach that combines clinical evaluation, standardized indices, endoscopic and histologic findings, laboratory biomarkers, and imaging to maximize diagnostic precision and appropriately classify disease phenotype before initiating therapy [1,24,25]. Noninvasive markers such as fecal calprotectin and CRP are increasingly incorporated into routine monitoring, enabling ongoing assessment of inflammation, guiding treatment decisions, and improving prognostication [1]. This comprehensive framework reflects the growing sophistication of contemporary IBD management, emphasizing precision and individualized care.

Table 2 summarizes the diagnostic features and activity indices used to evaluate UC and CD, facilitating standardized diagnosis and disease severity assessment.

**Table 2:** Diagnostic criteria and activity indices for inflammatory bowel disease UC: Ulcerative colitis; CD: Crohn's disease; PGA: Physician global assessment; SCCAI: Simple Clinical Colitis Activity Index; CDAI: Crohn’s Disease Activity Index; HBI: Harvey Bradshaw Index; IBDEX: Inflammatory Bowel Disease Index; CRP: C-reactive protein; ESR: Erythrocyte sedimentation rate; pANCA: Perinuclear antineutrophil cytoplasmic antibody; ASCA: Anti-*Saccharomyces cerevisiae* antibody; MRE: Magnetic resonance enterography; CTE: Computed tomography enterography; MRI: Magnetic resonance imaging.

Aspect	UC	CD	Diagnostic/Activity Indices and Notes
Clinical features	Persistent or recurrent mucous or bloody stools, increased stool frequency, and abdominal pain	Chronic abdominal pain, diarrhea (possibly bloody), weight loss, fever, and perianal lesions	Mayo Score (UC): Combines clinical symptoms, endoscopy, and PGA; SCCAI (UC): validated, correlates with biomarkers; CDAI (CD): composite of symptoms, labs, and complications; HBI (CD): simplified clinical tool
Endoscopic findings	Continuous mucosal inflammation beginning in the rectum, granularity, superficial ulceration, and pseudopolyps	Discontinuous (segmental) transmural lesions, deep longitudinal ulcers, cobblestoning, strictures, and fistulas	Endoscopic subscores are incorporated into the Mayo Score (UC).
Histopathological findings	Diffuse mucosal inflammatory infiltrates, crypt abscesses, and goblet cell depletion; chronic disease shows gland distortion and atrophy	Transmural inflammation with noncaseating granulomas, fissures, and fistulas	IBDEX: Proposed unified severity index for both UC and CD; preliminary validation only, not guideline-endorsed.
Laboratory tests	Elevated inflammatory markers (CRP, ESR); fecal calprotectin supports detection of mucosal inflammation; pANCA is more often positive in UC.	Same as UC, plus serologic markers (ASCA is more often positive in CD)	No single diagnostic test; labs are adjunctive for diagnosis, disease activity, and monitoring.
Imaging	Limited diagnostic role; barium enema may show “lead-pipe colon” in chronic disease	MRE and CTE detect small bowel inflammation with high sensitivity (>90%), and reveal strictures, fistulas, and penetrating disease	Imaging complements endoscopy; MRI refinements help distinguish fibrosis from inflammation and assess treatment response.

Pathophysiology of IBD

IBD, which includes CD and UC, is a long-lasting, recurring inflammatory condition of the gastrointestinal tract caused by an abnormal immune response to gut microbes in genetically susceptible individuals [26]. Defects in the intestinal epithelial barrier and environmental factors additionally interact to influence the onset, characteristics, and duration of disease [26]. Despite clinical and histological differences, CD and UC share a common pathway of chronic, dysregulated mucosal immune activation, ineffective inflammation resolution, and progressive intestinal damage [26]. CD causes transmural, discontinuous inflammation, while UC causes continuous, superficial mucosal inflammation [26,27].

Both innate and adaptive immune dysregulation contribute to the pathogenesis of IBD [28]. Genome-wide association studies (GWAS) have identified more than 200 risk loci associated with IBD, implicating diverse regulatory pathways [28]. These include cytokine signaling networks such as the IL-10 pathway and the IL-12/IL-23-T helper (Th) 1/Th17 axis (e.g., IL-23 receptor (IL23R)), as well as genes involved in autophagy (e.g., autophagy-related 16-like 1 (ATG16L1), immunity-related GTPase family M protein (IRGM)), microbial sensing (e.g., nucleotide-binding oligomerization domain-containing protein 2 (NOD2)), and epithelial barrier integrity (e.g., extracellular matrix protein 1 (ECM1)) [27,28]. Collectively, these genetic variants increase susceptibility to chronic intestinal inflammation by altering immune regulation and disrupting tolerance to commensal microorganisms [28]. In response, the intestinal epithelium leverages a variety of defense mechanisms, including immunoglobulin A (IgA) secretion, mucus production by goblet cells, tight junctions, and antimicrobial peptides like paneth-cell α-defensins to maintain a protective barrier and prevent microbial invasion [29]. Deterioration of primary or secondary tight-junction integrity, dysregulation of paneth-cell function (particularly in ileal CD), and a deficiency of the mucus layer that facilitates greater antigen access are all implicated in IBD generation by promoting immune activation and sustaining damage [29,30]. Reduced beneficial commensals and changed microbial metabolites that affect barrier integrity and immunity are among the qualitative and functional changes in the gut microbiome (also known as dysbiosis) that are characteristic of IBD [31]. A wider polybiome contribution is highlighted by the fact that, in addition to bacteria, the intestinal mycobiome and host antifungal pathways, such as dectin-1/C-type lectin domain family 7 member A (CLEC7A)-caspase recruitment domain-containing protein 9 (CARD9) signaling, interact with innate immunity and can alter the severity of colitis [31-33].

Signals from microbes and damage recognized by epithelial and myeloid pattern recognition receptors such as toll-like receptors (TLRs) and nucleotide-binding oligomerization domain-like receptors (NLRs) trigger complement activation, phagocytosis, and cytokine-chemokine cascades, resulting in increased neutrophil influx, activation of macrophages and dendritic cells, and impaired responses from innate lymphoid cells (ILCs), which are exacerbated by genetic issues in autophagy and microbial sensing [34]. Dendritic cells present antigens that subsequently initiate adaptive immune responses skewed toward Th1/Th17 pathways driven by interferon-gamma (IFN-γ), IL-17, and IL-22 under the influence of IL-12/23 in CD while prompting a nonclassical Th2-like profile (e.g., IL-13) with the participation of ILC2/ILC3 in UC [35].

The pathophysiology and clinical variability of IBD are significantly influenced by environmental exposures [36]. Smoking appears to be protective in UC, lowering incidence and attenuating disease activity, but it also increases the risk and severity of CD, with higher rates of stricturing, fistulization, and postoperative recurrence [37]. Cigarette smoke and nicotine change mucosal immunity, impair microvascular flow, disrupt mucus and epithelial permeability, and induce oxidative stress [38]. This creates an environment that promotes transmural inflammation in CD but may reduce superficial colonic inflammation in UC [38]. Another consistent risk factor is antibiotic exposure, especially when it is repeated, broad-spectrum, or given early in life [39]. This exposure links to new-onset IBD and disease flares by reducing beneficial bacteria such as *Bifidobacteria *and butyrate producers; this decrease reduces short-chain fatty acid (SCFA) production and allows harmful bacteria to thrive [39]. Nutrition also plays a role; diets high in saturated fat, animal protein, and processed food additives, common in Western diets, are associated with a higher incidence of IBD [40]. In contrast, high-fiber or Mediterranean diets relate to a lower risk [40]. These dietary effects happen through changes in microbial diversity and metabolite production, particularly SCFAs, altered bile acid levels, and direct effects on barrier integrity and innate immune signaling [40,41].

Persistent inflammation leads to sustained activation and proliferation of myofibroblasts, altering the mucosa in CD [42]. In CD, transmural inflammation promotes the formation of fissures, fistulas, and strictures through myofibroblast activation and excessive extracellular matrix deposition [42]. In contrast, in UC, inflammation is typically confined to the mucosa and submucosa, resulting in superficial but continuous lesions throughout the colon [42]. Persistent cytokine and chemokine signaling is associated with epithelial ulceration and crypt structural distortion in UC, along with fibrostenotic changes in CD [43]. Impaired removal of microorganisms and dying cells, persistent pattern recognition receptor (PRR) signaling, and a lack of pro-resolving mediators all play a role in sustaining IBD by obstructing resolution pathways [44]. The result is ongoing tissue remodeling, repeated injury, and insufficient recovery, all features of chronic, relapsing inflammation [43,44].

**Figure 2:**
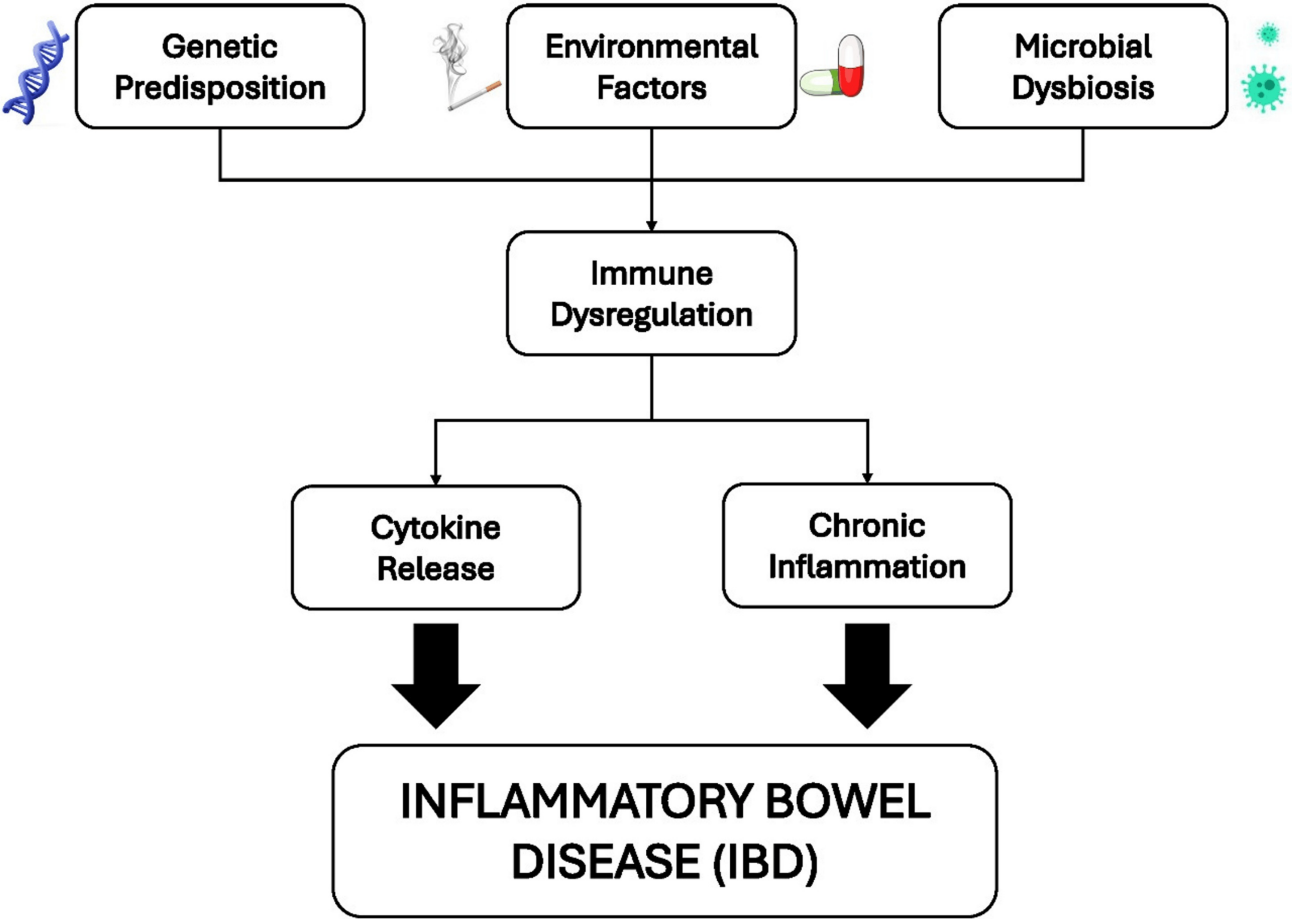
Pathophysiology of IBD Image credits: This image was created by Tabish W Siddiqui. IBD: Inflammatory bowel disease.

Figure 2 elucidates the various factors involved in the pathophysiology of IBD.

Clinical insights into arrhythmias in IBD

IBD-Related Arrhythmia

IBD is increasingly recognized as a systemic inflammatory disorder with substantial cardiovascular implications, including acute myocardial infarction (AMI), heart failure (HF), cerebrovascular events, and a wide spectrum of arrhythmias [6]. AF is the most frequently reported, though supraventricular tachycardia (SVT), ventricular tachycardia (VT), and conduction abnormalities such as atrioventricular block (AVB) are also observed [45]. Chronic systemic inflammation is the primary driver of the arrhythmogenic risk in IBD, and elevated levels of cytokines such as TNF-α, IL-6, and CRP disrupt ion channel function, altering calcium handling, and impairing gap junction communication [46]. These changes, compounded by autonomic dysfunction with increased sympathetic tone, promote abnormal conduction and heightened excitability. Additionally, myofibroblasts and matrix metalloproteinases are activated by inflammatory mediators, resulting in cardiac fibrosis and structural remodeling that produces substrates for reentrant circuits [47]. These processes are exacerbated by immune cell infiltration during IBD flares, and subclinical changes such as atrial electromechanical delay have been demonstrated even in adolescent patients, underscoring the systemic cardiovascular impact of IBD [46].

Beyond inflammation, IBD patients face additional arrhythmogenic risk factors. Electrolyte disturbances such as hypokalemia, hypomagnesemia, and hypocalcemia, often resulting from malabsorption and chronic diarrhea, may predispose to conduction abnormalities [48]. Anemia, another common complication, increases myocardial oxygen demand and workload, further destabilizing cardiac function [49]. The convergence of inflammatory, autonomic, metabolic, and structural mechanisms highlights the need for comprehensive cardiovascular assessment and monitoring in this population to enable timely detection and management of arrhythmias and related complications. Table 3 elucidates the relationship between IBD and arrhythmias, as observed in various studies.

**Table 3:** Association between IBD and cardiovascular outcomes GWAS: Genome-wide association study; IBD: Inflammatory bowel disease; UC: Ulcerative colitis; AVB: Atrioventricular block; CD: Crohn's disease; HR: Hazard ratio; AF: Atrial fibrillation; SVT: Supraventricular tachycardia; VT: Ventricular tachycardia; IBD-U: Inflammatory bowel disease-unclassified; aHR: Adjusted hazard ratio; CI: Confidence interval; AMI: Acute myocardial infarction; HF: Heart failure.

Reference	Study	Study Design	Population Size	Participants	Statistical Findings	Type Of Arrhythmia/ Cardiac Event	Key Findings	Result Summary
Shu and Huang, 2025 [9]	Mendelian Randomization Study	Genetic phenotype analysis	GWAS: 34,652 IBD (12,882 cases, 21,770 controls); UC: 27,432 (6,968 cases, 20,464 controls)	Genetically analyzed European Ancestry IBD patients	p-value = 0.000828 (UC and AVB)	AVB, paroxysmal tachycardia	UC significantly increases the risk of AVB; no causal association found between IBD or CD and paroxysmal tachycardia.	Strong genetic evidence UC increases AVB risk independent of confounders/drugs; clinical implications for proactive cardiac monitoring and management
Narous et al., 2025 [10]	University of Manitoba Cohort Study	Retrospective cohort study	10,992 IBD patients vs 102,875 controls	Adult IBD patients matched to controls	HR 1.51 for arrhythmia risk	AF, SVT, VT	IBD patients have a significantly higher risk of AF, SVT, and VT, risk independent of prior heart disease.	Younger patients showed increased arrhythmia risk.
Sun et al., 2023 [11]	Population-Based Long-Term Arrhythmia Study	Sibling-controlled cohort study	83,877 IBD patients: CD (24,954), UC (46,856), IBD-U (12,067) plus matched controls and IBD-free siblings	Biopsy-confirmed IBD patients and matched controls and siblings in Sweden, 1969–2017	CD aHR 1.15 (95% CI: 1.09–1.21) UC aHR 1.14 (95% CI: 1.10–1.18) IBD-U aHR 1.30 (95% CI: 1.20–1.41)	AF/flutter, SVT, VT	IBD is associated with an increased risk of overall and specific arrhythmias, persisting 25 years post-diagnosis.	IBD is associated with a significantly increased long-term risk of overall and specific arrhythmias, persisting up to 25 years; sibling comparison confirms findings; no increased risk for bradyarrhythmias.
Aniwan et al., 2018 [12]	Olmsted County Cohort Study	Population-based cohort	736 IBD patients and 1,472 matched controls	Adult patients from Olmsted County	AMI aHR 2.82 (95% CI: 1.98–4.04); HF aHR 2.03 (95% CI: 1.36–3.03)	AMI, HF	IBD patients have nearly three times higher risk of AMI and twice the risk of HF vs matched controls, despite lower prevalence of traditional CV risk factors; UC and CD both showed elevated AMI risk; UC showed increased HF risk.	Elevated risk of AMI and HF in IBD patients

In a Mendelian randomization study by Shu et al. (2025), strong causal genetic evidence that UC increases the risk of AVB, with an OR of 1.178 (95% CI: 1.070-1.297, p = 0.000828) was established [9]. The contribution of UC to conduction problems is uniquely isolated by this evidence, which is less susceptible to bias than observational research and unaffected by outside variables like medication use. The arrhythmia phenotype specificity is further refined by the absence of a causative relationship between IBD or CD and paroxysmal tachycardia. This fosters targeted clinical vigilance for AVB in UC patients.

Supporting these genetic insights, the University of Manitoba retrospective cohort by Narous et al. (2025) found that IBD patients had a significantly elevated risk of cardiac arrhythmias (HR: 1.51, 95% CI: 1.30-1.76), including AF, SVT, and VT, independently of prior heart disease [10]. Interestingly, this large cohort demonstrated that common IBD medications such as 5-ASA, thiopurines, and TNF-α inhibitors were not associated with arrhythmia risk, implicating disease-related inflammatory processes as the predominant factors. The younger median age at IBD diagnosis (35 years) and increased arrhythmia risk in younger subgroups emphasize the need for early cardiovascular risk screening.

Extending this, Sun et al. (2023) conducted a nationwide Swedish sibling-controlled cohort with 83,877 IBD patients and demonstrated that the elevated risk of overall and specific arrhythmias (including AF/flutter, other SVT, and ventricular arrhythmias/cardiac arrest) persists up to 25 years post-diagnosis [11]. The adjusted hazard ratios (aHR) ranged from 1.14 (UC) to 1.30 (IBD-unclassified), all highly significant (p < 0.001). The sibling comparison controlled for shared genetic and environmental confounders, reinforcing the IBD-arrhythmia link beyond familial factors. Notably, no significant association was observed for bradyarrhythmias, which aligns with Shu and Huang's findings specifying AVB risk limited to UC [9].

The Olmsted County cohort by Aniwan et al. (2018) complements these arrhythmia studies by highlighting the elevated risk of hard cardiovascular outcomes like AMI and HF in IBD patients [12]. The aHR for AMI (2.82; 95% CI: 1.98-4.04) and HF (2.03; 95% CI: 1.36-3.03) were notably high, underscoring the systemic cardiovascular burden. Unlike the arrhythmia-focused studies, Aniwan et al. contextualized IBD as a potent risk factor for ischemic and functional myocardial disease, reinforcing the concept that chronic inflammation in IBD produces widespread cardiac consequences.

While Narous et al. and Sun et al. report increased risks of multiple arrhythmia types including AF, SVT, and VT, the Mendelian randomization study by Shu et al. specifically implicates UC in conduction disorder risk but not tachyarrhythmias, suggesting subtype-specific arrhythmogenic substrates [9-11]. The Manitoba cohort’s lack of medication effect on arrhythmia risk contrasts with other literature suggesting corticosteroids may raise cardiovascular risk, highlighting the complex interplay between disease activity, therapy, and arrhythmogenesis [10].

These studies’ effect sizes vary but generally indicate modest to moderate increased risks (HRs from ~1.1 to nearly 3), highlighting clinically meaningful but non-universal susceptibility. Notably, the long-term persistence of arrhythmia risk demonstrated up to 25 years by Sun et al. emphasizes that cardiovascular monitoring in IBD requires sustained attention well beyond initial diagnosis, with the sibling-controlled design strengthening causal inference amid potential confounding [11]. Although each investigation addresses different aspects of this relationship, collectively they reveal a consistent pattern of heightened cardiovascular vulnerability in IBD, refined by disease subtype, arrhythmia phenotype, and long-term disease trajectory. These findings further suggest that systemic inflammation intrinsic to IBD, rather than conventional risk factors or therapies, plays a central role in the development of arrhythmogenic and myocardial complications.

Medication-Related Arrhythmia

Recent clinical studies and pharmacovigilance analyses yield complementary insights into the cardiovascular safety profiles of IBD treatments, highlighting different mechanistic characteristics among 5-ASA, corticosteroids, and biologic medications [8]. Key studies summarizing 5-ASA-associated myocarditis, corticosteroid effects, and anti-TNF cardiovascular safety are summarized in Table 4.

**Table 4:** Cardiovascular safety profiles of major IBD therapies POAF: Postoperative atrial fibrillation; RCT: Randomized controlled trial; RR: Relative risk; CI: Confidence interval; CABG: Coronary artery bypass graft; GI: Gastrointestinal; TNF: Tumor necrosis factor; IBD: Inflammatory bowel disease; PY: Person-years; CD: Crohn's disease; UC: Ulcerative colitis; HR: Hazard ratio; WHO: World Health Organization; UKCRPD: United Kingdom Clinical Practice Research Datalink; RA: Rheumatoid arthritis; SLE: Systemic lupus erythematosus; PMR: Polymyalgia rheumatica; AF: Atrial fibrillation; HF: Heart failure; MI: Myocardial infarction; CVD: Cardiovascular disease; IV: Intravenous.

Reference	Study	Study Design	Population Size	Participants	Statistical Findings	Type of Arrhythmia/Cardiac Event	Key Findings	Result Summary
Zhou et al., 2023 [13]	Glucocorticoids and POAF	Meta-analysis of RCT	27 studies; 14,442 patients	Cardiac surgery patients receiving glucocorticoids vs control	RR = 0.80 (95% CI: 0.71–0.92, p = 0.001); low-dose RR = 0.81 (95% CI: 0.71–0.92); high-dose not significant; CABG subgroup RR = 0.71 (95% CI: 0.58–0.87)	POAF	Low-dose glucocorticoids significantly reduced POAF incidence, especially in CABG; no increase in infection or GI complications	Glucocorticoids significantly reduce POAF risk in CABG patients; low doses are more effective; no rise in infection or GI injury.
Larsen et al., 2023 [14]	Anti-TNF treatment and cardiac arrhythmia risk in IBD	Nationwide population-based cohort study	44,053 IBD patients; 379,862 PY follow-up; CD (14,660) and UC (29,393)	IBD patients exposed vs unexposed to anti-TNF (infliximab, adalimumab, golimumab)	HR = 0.95 (95% CI: 0.80–1.13); sensitivity HR = 1.10 (95% CI: 0.85–1.42); no sex-specific differences	Any cardiac arrhythmia, cardiac arrest	Anti-TNF therapy not linked to increased arrhythmia risk; reassuring cardiac safety profile	Large national data show no increased arrhythmia risk with anti-TNF; long-term findings support cardiovascular safety.
Nguyen et al., 2022 [15]	Pharmacovigilance analysis (WHO VigiBase)	Worldwide observational cross-sectional study	21 million safety reports; 5,108 myocarditis cases	Worldwide reports of myocarditis linked to various drugs	62 drugs identified; 41 in 5 classes; mortality 10.3% overall; immunotherapy-related 32.5%; median onset 15 days	Myocarditis	Myocarditis associated with mesalazine and sulfasalazine; onset ~15 days; fatality is highest for immunotherapies.	Mesalazine and sulfasalazine are associated with myocarditis.
Chen et al., 2022 [16]	Mesalazine-induced cardiotoxicity in IBD	Systematic review	52 cases (40 males, 12 females)	IBD patients receiving mesalazine	Median onset 14 days; 82.7% chest pain; 46.2% fever; 40.4% dyspnea; 10.3% mortality	Myocarditis, pericarditis	Mesalazine can cause myocarditis and pericarditis; prompt discontinuation leading to cessation; corticosteroids may help.	Mesalazine-related myocarditis is rare, but potentially fatal; early recognition and drug discontinuation are essential.
Liu et al., 2021 [17]	Corticosteroid use and POAF prevention after cardiac surgery	Meta-analysis of RCTs	14 RCTs; 13,803 patients	Cardiac surgery patients randomized to corticosteroids vs control	RR = 0.70 (95% CI: 0.55–0.89, p = 0.003); no significant difference in infection (RR = 1.01), mortality (RR = 0.87), or GI complications (RR = 1.26)	POAF	Corticosteroids reduce POAF risk without increasing infection or mortality	Corticosteroids reduce POAF without leading to major complications.
Pujades-Rodriguez et al., 2020 [18]	UKCRPD	Population-based cohort study	87,794 patients; 27,739 with IBD	Six immune-mediated inflammatory diseases (IBD, RA, SLE, PMR, vasculitis)	HR for <5 mg prednisolone: 1.74 (95% CI: 1.64–1.84); AF HR 1.69; HF HR 1.75; AMI HR 1.76	AF, HF, MI	Even low-dose glucocorticoids increase AF and CVD risk regardless of disease activity	Even low-dose glucocorticoids increase CVD risk; this underscores the need for cardiac monitoring during prolonged use.
Halonen et al., 2007 [19]	Hydrocortisone and prevention of AF after cardiac surgery	Double-blind randomized multicenter trial	241 patients	CABG, aortic valve, or combined surgery without prior AF	POAF incidence 30% (hydrocortisone) vs 48% (placebo); HR = 0.54 (95% CI: 0.35–0.83, p = 0.004)	POAF	IV hydrocortisone reduces POAF incidence without increasing infection or major complications.	Hydrocortisone significantly lowers POAF rates; effective and well-tolerated anti-inflammatory prophylaxis.

Biologic therapy, particularly anti-TNF agents, demonstrates a reassuring cardiovascular safety profile. Larsen et al. (2023) conducted a nationwide population-based cohort study of 44,053 IBD patients (379,862 person-years of follow-up) and found no increase in arrhythmia risk among anti-TNF users compared with unexposed controls (HR 0.95; 95% CI: 0.80-1.13, p = 0.79) [14]. Sensitivity analyses and subgroup assessments showed consistent neutrality across sex and IBD subtype, supporting the cardiac safety of infliximab, adalimumab, and golimumab [14]. These observations are consistent with findings from the Narous et al. (2025) cohort, which further supports the lack of arrhythmogenic risk associated with common IBD therapies [10]. Isolated case reports of infusion-related arrhythmias likely represent transient reactions rather than sustained electrophysiologic toxicity.

The 5-ASA-related cardiotoxicity remains a well-recognized but uncommon adverse effect. Nguyen et al. (2022) examined more than 21 million records in the WHO VigiBase, pinpointing mesalazine and sulfasalazine as the predominant medications associated with myocarditis among IBD treatments, typically emerging within two weeks of initiation [15]. The uniformity among various populations highlights a reliable pharmacovigilance trend linking 5-ASA exposure to hypersensitivity-related cardiac inflammation. In addition to these extensive findings, Chen et al. (2022) conducted a systematic review and found 52 documented instances of cardiotoxicity induced by mesalazine, revealing a notably uniform clinical phenotype that emerged within about two weeks of starting therapy, with the main symptoms being myocarditis or pericarditis, and nearly all cases resolved following the cessation of the drug [16]. The reproducibility, reversibility, and eosinophilic histopathology strongly support a biologically plausible, immune-mediated process [16]. Collectively, these studies strongly confirm mesalazine myocarditis as a reproducible, hypersensitivity-mediated, and reversible complication specifically linked to 5-ASA treatment [16].

Corticosteroids demonstrate a clear time and dose-dependent duality in cardiovascular outcomes. Research from randomized controlled trials (RCTs) and meta-analyses, such as Halonen et al. (2007), Liu et al. (2021), and Zhou et al. (2023), consistently shows that short-term perioperative corticosteroid use significantly decreases the incidence of postoperative atrial fibrillation (POAF) following cardiac surgery [13,17,19]. Resál et al. found a 30% occurrence of AF with hydrocortisone, in contrast to 48% in the placebo group (HR 0.54; p = 0.004) [49]. While Liu et al. confirmed a similar benefit (RR 0.70; 95% CI: 0.55-0.89; p = 0.003) without excess infection or mortality [17], Zhou et al. further demonstrated that low-dose glucocorticoids conferred maximal benefit, particularly among coronary artery bypass graft (CABG) patients (RR 0.71; 95% CI: 0.58-0.87) [13]. These findings highlight transient anti-inflammatory and antiarrhythmic properties mediated by cytokine suppression and attenuation of atrial remodeling. In contrast, chronic glucocorticoid exposure yields opposite effects [18]. The population-based study by Pujades-Rodriguez et al. (2020) involving 87,794 patients (including 27,739 with IBD) revealed a dose-dependent increase in AF risk, even with <5 mg/day of prednisolone (adjusted HR 1.69; 95% CI: 1.54-1.85) [18]. The difference between brief protective effects and long-term detrimental effects highlights the opposing physiological consequences of temporary immunosuppression compared to prolonged glucocorticoid-induced hypertension, sympathetic activation, and electrolyte imbalance.

In summary, these studies identify three unique pharmacologic cardiac profiles among IBD treatments. Initially, 5-ASA agents are distinctly linked to hypersensitivity-mediated myocarditis, a reversible and reproducible condition validated by both pharmacovigilance and clinical data [15,16]. Second, corticosteroids show a time-dependent duality, wherein short-term, low-dose administration is antiarrhythmic by reducing inflammation, whereas long-term use can enhance arrhythmia development due to systemic metabolic and hemodynamic impacts [13,17,19,50]. Third, anti-TNF biologics show cardiac neutrality, exhibiting no significant increase in arrhythmia risk compared to unexposed controls [14]. The consistency of these results across diverse datasets highlights the complex relationship between immunomodulation and cardiovascular safety in IBD treatment, stressing the significance of exposure context, acute versus chronic, in assessing risk [51].

Management

Effective management of IBD in the context of arrhythmic and cardiovascular risk requires a coordinated, pathophysiologically informed strategy targeting both intestinal inflammation and systemic cardiovascular vulnerability [52]. Baseline cardiovascular evaluation, including medical history, physical examination, ECG, and laboratory assessment of electrolytes, hemoglobin, and inflammatory biomarkers, should be integrated into routine IBD care, particularly in patients with prolonged disease or metabolic comorbidities [1]. In moderate to severe cases, echocardiography may identify subclinical dysfunction [52]. Therapeutic selection must balance anti-inflammatory efficacy with cardiac safety [52].

Effective control of mucosal and systemic inflammation is central to reducing arrhythmogenic risk in IBD, as proinflammatory cytokines, notably TNF-alpha and IL-6, modulate cardiac electrophysiology by altering calcium handling and connexin-mediated conduction, increasing atrial excitability and conduction heterogeneity [35]. For mild to moderate UC, aminosalicylates such as mesalazine or sulfasalazine remain foundational therapies, though rare hypersensitivity-related myocarditis or pericarditis can occur early and typically resolve after drug withdrawal [53]. In CD, however, aminosalicylates play a lesser role, with corticosteroids, immunomodulators, and biologics constituting the mainstay of therapy [7]. In moderate to severe UC or CD, immunomodulators and biologics are preferred for long-term maintenance due to superior inflammation control and favorable cardiovascular safety, with anti-TNF agents and newer biologics, including vedolizumab and ustekinumab, demonstrating no increased arrhythmia risk and potential indirect cardioprotection through systemic inflammation suppression [54]. Chronic corticosteroid use exerts dose-dependent cardiovascular effects, including hypertension, metabolic dysregulation, and electrolyte imbalance, despite transient antiarrhythmic benefits, necessitating restriction to short induction courses with transition to steroid-sparing agents and ECG and blood pressure monitoring for prolonged therapy [13].

Recent advances in IBD therapeutics are transforming management by enabling effective control of systemic inflammation while addressing cardiovascular safety. Janus kinase inhibitors (JAKinibs), including tofacitinib and upadacitinib, demonstrate efficacy in moderate to severe UC and CD, with studies showing no statistically significant increase in major adverse cardiovascular events, venous thromboembolism, or overall cardiovascular events, although dose-dependent trends for higher doses warrant caution [55]. Selective tyrosine kinase 2 (TYK2) inhibitors, such as deucravacitinib, appear to offer a favorable cardiovascular profile with reduced thrombosis and arrhythmic risk [55]. Given the potential effects on lipid metabolism and thrombosis, baseline cardiovascular risk assessment, lipid profiling, and periodic ECG monitoring are recommended during therapy. Emerging gut-selective biologics targeting integrins, chemokine receptors, and T-cell trafficking minimize systemic exposure and reduce cardiovascular risk [56]. Vedolizumab, targeting alpha-4 beta-7 (α4β7) integrin, shows high gut selectivity, minimal immunosuppression, and consistently favorable cardiovascular safety [54,56]. Complementary microbiome-directed therapies, including fecal microbiota transplantation and precision probiotics, may restore microbial balance, reduce systemic inflammation, and attenuate arrhythmogenic remodeling [57]. Experimental research on specialized pro-resolving lipid mediators, such as resolvins, protectins, and maresins, indicates potential to limit systemic inflammatory spillover, support durable mucosal healing, and mitigate arrhythmogenic remodeling [58]. Collectively, these developments reflect a shift toward precision therapeutics in IBD that balance intestinal efficacy with extraintestinal safety, emphasizing individualized cardiovascular risk assessment, monitoring, and judicious therapy selection to optimize long-term cardiac outcomes while maintaining disease remission.

Long-term cardiovascular surveillance is essential in IBD management, as studies show persistently elevated arrhythmia risk independent of traditional cardiovascular factors [9]. Annual ECG screening is advised for patients with active disease, prior arrhythmias, or prolonged corticosteroid use, with ambulatory monitoring for symptomatic individuals [13]. Maintaining electrophysiologic stability requires correcting electrolyte imbalances, optimizing nutrition, treating anemia, and ensuring adequate hydration [59]. Multidisciplinary coordination between gastroenterologists, cardiologists, and internists ensures cohesive therapy and monitoring [59]. Patient education on recognizing cardiac warning signs, adhering to treatment, and adopting cardioprotective lifestyle measures such as smoking cessation, regular exercise, and a Mediterranean-style diet reduces systemic inflammation and cardiovascular burden [59]. Effective management of arrhythmia risk in IBD integrates inflammation control, careful therapy selection, and ongoing cardiovascular monitoring. Incorporating cardiovascular endpoints into clinical trials and longitudinal registries, alongside personalized patient stratification, will be critical to developing strategies that safeguard both intestinal and cardiac health in this multi-systemic disease [60].

Limitations and Future Directions

Although current research has provided valuable insights into the link between IBD and arrhythmogenesis, several limitations constrain the robustness and generalizability of existing evidence. Much of the literature remains observational, retrospective, or registry-based, restricting causal inference and leaving findings susceptible to confounding from disease activity, comorbidities, and medication use. Heterogeneity in disease phenotyping, arrhythmia classification, and diagnostic criteria further complicates direct comparison and meta-analytical synthesis. Treatment-stratified and activity-adjusted analyses are limited, with few studies accounting for varying inflammation levels or the timing of biologic and immunomodulatory therapies. Cardiovascular outcomes are often secondary endpoints, captured through administrative codes rather than standardized ECG monitoring, risking misclassification. Mechanistic investigations remain sparse, limiting understanding of the cellular and electrophysiologic pathways linking systemic inflammation to conduction disturbances. Underrepresentation of diverse populations and a paucity of long-term follow-up highlight the need for rigorous, prospective, cardiovascular-focused research.

Future studies should prioritize integrative, longitudinal designs that simultaneously assess disease activity, genetic susceptibility, and therapy exposure in relation to arrhythmia risk. Advanced imaging and continuous ECG monitoring may illustrate causative links between chronic intestinal inflammation and myocardial remodeling. Precision medicine approaches that stratify patients by molecular phenotype, cytokine profile, and cardiac risk could guide tailored monitoring and therapy. International registries with standardized diagnostic and arrhythmia definitions would strengthen comparability and causal inference. Randomized trials and pharmacovigilance analyses should systematically evaluate cardiovascular endpoints across emerging IBD therapies. Multidisciplinary care pathways involving gastroenterology, cardiology, and immunology can facilitate early detection and management of cardiovascular complications. Integrating these insights into clinical algorithms balancing inflammation control and cardiovascular safety will be critical for optimizing long-term outcomes in IBD.

## Conclusions

IBD is increasingly recognized as a systemic disorder with important cardiovascular consequences. Evidence consistently indicates that chronic inflammation contributes to a higher risk of cardiac arrhythmias, independent of traditional risk factors. While therapeutic agents such as corticosteroids and aminosalicylates may exert variable cardiovascular effects, biologic therapies appear largely safe and may even mitigate arrhythmic risk by controlling systemic inflammation. A comprehensive approach that includes routine cardiovascular evaluation, optimized inflammation control, and multidisciplinary collaboration is essential for improving patient outcomes. Future research should aim to clarify the molecular pathways linking intestinal and cardiac inflammation and to establish standardized monitoring and prevention strategies. These efforts will be crucial in reducing the long-term cardiovascular burden among individuals with IBD.

## Appendices

Appendix 1: Full search strategy for each database

PubMed/MEDLINE

(("Inflammatory Bowel Disease"[Mesh] OR "Crohn Disease"[Mesh] OR "Ulcerative Colitis"[Mesh]

OR "Inflammatory Bowel Disease"[tiab] OR "Crohn's Disease"[tiab] OR "Ulcerative Colitis"[tiab])

AND

("Arrhythmias, Cardiac"[Mesh] OR "Atrial Fibrillation"[Mesh] OR "Ventricular Tachycardia"[Mesh]

OR "Arrhythmia"[tiab] OR "Atrial Fibrillation"[tiab] OR "Ventricular Tachycardia"[tiab])

AND

("Drug Therapy"[Mesh] OR "IBD treatment"[tiab] OR "biologics"[tiab] OR "corticosteroids"[tiab] OR "JAK inhibitors"[tiab]))

Filters: English, Publication dates 2000/01/01 to 2025/09/25

SCOPUS

(TITLE-ABS-KEY("Inflammatory Bowel Disease" OR "Crohn's Disease" OR "Ulcerative Colitis"))

AND

(TITLE-ABS-KEY("Arrhythmia" OR "Atrial Fibrillation" OR "Ventricular Tachycardia"))

AND

(TITLE-ABS-KEY("IBD treatment" OR "biologics" OR "corticosteroids" OR "JAK inhibitors"))

AND (LIMIT-TO(LANGUAGE, "English")) AND (PUBYEAR > 1999 AND PUBYEAR < 2026)

Embase

('inflammatory bowel disease'/exp OR 'crohn disease'/exp OR 'ulcerative colitis'/exp

OR 'inflammatory bowel disease':ti,ab OR 'crohn\'s disease':ti,ab OR 'ulcerative colitis':ti,ab)

AND

('arrhythmia'/exp OR 'atrial fibrillation'/exp OR 'ventricular tachycardia'/exp

OR 'arrhythmia':ti,ab OR 'atrial fibrillation':ti,ab OR 'ventricular tachycardia':ti,ab)

AND

('drug therapy'/exp OR 'IBD treatment':ti,ab OR 'biologics':ti,ab OR 'corticosteroids':ti,ab OR 'JAK inhibitors':ti,ab)

AND (english)/lim AND (2000-2025)/py

Google Scholar

("Inflammatory Bowel Disease" OR "Crohn's Disease" OR "Ulcerative Colitis")

AND

("Arrhythmia" OR "Atrial Fibrillation" OR "Ventricular Tachycardia")

AND

("IBD treatment" OR "biologics" OR "corticosteroids" OR "JAK inhibitors")

Filters: Articles from 2000-2025, English language
